# Orienting field effects on the flow of an active nematic liquid crystal in a channel

**DOI:** 10.1140/epje/s10189-025-00527-x

**Published:** 2025-11-10

**Authors:** Joshua Walton, Geoffrey McKay, Nigel J. Mottram

**Affiliations:** 1 School of Mathematics and Statistics, University of Glasgow, University Place, Glasgow, G12 8QQ UK; 2 Department of Mathematics and Statistics, University of Strathclyde, 26 Richmond Street, Glasgow, G1 1XH UK

## Abstract

**Abstract:**

We examine the influence of an external orienting field on the director orientation and fluid flow of an active nematic liquid crystal confined in a channel, subject to infinite anchoring of the director and no-slip conditions at the channel walls. A mathematical model based on the Ericksen–Leslie dynamic equations for nematic liquid crystals is employed, with an additional active stress tensor accounting for the activity of the fluid. By solving the fully coupled nonlinear equations numerically, we investigate the dynamic response and the steady state of the active nematic when an orienting field is switched on. The dynamic behaviour when an orienting field is switched off is also examined, with our model demonstrating how the activity of the liquid crystal can enhance or hinder the classically observed kickback immediately after switch-off and generate nontrivial steady-state solutions. Specifically, we find that kickback, which can delay relaxation of the system to a steady state, can be made less pronounced, and eventually completely avoided, for contractile agents with a high activity parameter, even with a high magnitude orienting field value.

**Graphical abstract:**

Region of kickback effect in the space of activity and orienting field parameters - increased contractile behaviour will delay kickback to higher orienting field values
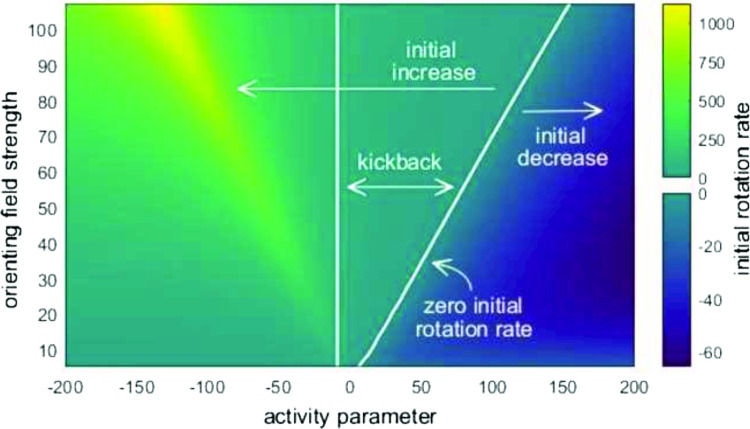

## Introduction

Active nematic liquid crystals are anisotropic fluids consisting of flow-generating agents that contain a supply of internal energy, such as in suspensions of bacteria [1–3], swimming organisms [4, 5], and cytoskeletal filaments such as microtubule networks [6, 7]. Such systems allow for collective orientational ordering and flow-orientation coupling similar to nematic liquid crystals, as well as spontaneous flow generation [8–13] that produces an out-of-equilibrium system [14, 15]. Unlike classical molecular (inactive) nematics, which consist of rod-like or disc-like nanometre-scale molecules, the constituent units of active fluids span subcellular micron-length scales or larger (for example, microtubules and bacterial suspensions, respectively). In all cases, the active agents in such systems exert stresses on either a background fluid or directly on other agents. The stresses can depend on the orientation of agents, for instance, defined by the long axis of the bacterium or microtubule, and the macroscopic symmetry of the liquid-crystalline-like phase. Internally generated active stress can lead to a wide array of interesting effects, including hydrodynamic and distortional instabilities, microchannel pumping, and the creation and annihilation of defects and multiscale flows reminiscent of turbulence [16–28].

The similarities between the orientational ordering of active agents in a fluid and elongated rod-like molecules of a nematic liquid crystal mean that continuum hydrodynamic models of nematics have commonly been adopted in the theoretical modelling of active nematics [3, 29]. One such model, which we consider in this article, is based on the Ericksen–Leslie dynamic equations for nematic liquid crystals first proposed by Ericksen [30] and completed by Leslie [31–33]. These nonlinear, partial differential equations couple the dynamics of the director orientation to the fluid velocity and pressure and are derived from the conservation laws of mass, linear momentum and angular momentum. To account for effects due to activity, the Ericksen–Leslie equations are modified via additional terms that drive the system out of thermodynamic equilibrium [1, 3, 10]. This approach of including nonequilibrium terms in hydrodynamic models of liquid crystals has also been used in the theoretical modelling of other phases of active fluids, including active cholesteric liquid crystals [34–40] and active polar fluids [8, 41–47].

The potential for applications of active fluid systems in different industries has received attention from experimentalists and theoreticians in recent years, with a focus on the role of confinement [48–53] and methods of controlling the behaviour of active agents [54–60]. One technique, which has been commonly adopted to control and design active fluid systems and forms the basis of the work in this article, is the application of an external orienting field (such as light, magnetic or electric fields) [54, 61–67]. The use of an orienting field to control inactive nematics is nearly a century old and now forms the basis for ubiquitous liquid crystal displays (LCDs) present in many electronic devices [69–71, 75]. In such devices, an orienting field competes with surface orienting effects and, if the field strength passes a critical value, leads to a reorientation of the average molecular direction, known as the Freedericksz transition, and a consequential optical effect. Research undertaken by Pieranski et al. [76] on the dynamics of the Freedericksz transition in nematic liquid crystals demonstrated that changes in the director orientation due to an orienting field can induce flow. In some cases, the flow generated in liquid crystals by director reorientation, known as backflow, may cause a reduction in the effective rotational viscosity of the liquid crystal leading to faster switching times or novel domain switching mechanisms [71–74]. However, more usual is a delay in director orientation relaxation, with the transient induced flow after the removal of an orienting field leading to a reverse rotation of the director before a relaxation back to the zero-field director configuration [76–78]. The presence of this kickback of the director can have practical implications for the switching of nematic liquid crystal devices, potentially increasing switch-off times. By solving a decoupled version of the Ericksen–Leslie equations, Mottram et al. [79] predicted an effective rotational viscosity and demonstrated how kickback can be avoided, and the switch-off time optimised, by restricting the applied voltage to an appropriate range. This followed from the work in [78, 80] which, through a linearisation of the Ericksen–Leslie equations, proved analytically that the first-order mode for the director orientation for a similar setup as that in [79] will always exhibit kickback when a sufficiently large field is switched off.

In active nematics, the coupling between an orienting field and flow is less explored. However, experiments undertaken by Guillamant et al. [54] have shown that the application of a uniform magnetic field to an aqueous gel, consisting of micron-sized microtubules, allows for the active nematic to undergo a transition between turbulent and laminar flow regimes. Dervaux et al. [61] considered a theoretical model of how swimming microorganisms respond to the application of an orienting field in the form of a light source. Microorganisms tended to move towards areas of high light intensity, and the flow generated led to instabilities in the form of travelling waves. Rajabi et al. [64] conducted experiments which investigated how the speed and direction of active droplets in a nematic cell could be controlled using a focussed laser beam and an electric field. In that work, it was shown that when an electric field is switched on, the speed of the active droplets is reduced due to reorientation towards the direction of the electric field. When the electric field was switched off, the active droplets exhibited a steady unidirectional flow. Electric fields have also been shown to be able to control the motion of defects in an active nematic consisting of asymmetric colloidal particles [67]. Kinoshita et al. [68] demonstrated that director reorientation in active nematics due to spatially uniform electric fields can cause anisotropic active turbulence, characterised by enhanced flow perpendicular to the electric field. Our primary objective in this article is to investigate the effects of an orienting field on the flow of active nematics and compare to the effects observed in inactive nematics.

**Fig. 1:**
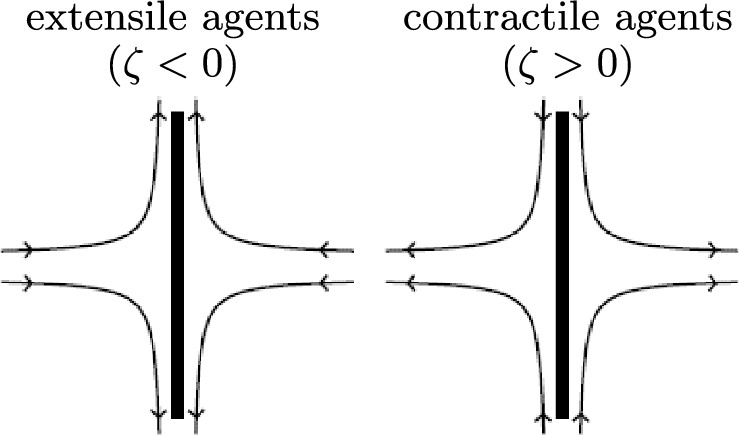
A schematic illustration of the flow induced by extensile (pusher) and contractile (puller) active agents, with arrows indicating the direction of the flow around the long axes of the active agent, indicated by the central thick solid line

As is now common, we consider a continuum theory of active nematics which accounts for the flow-generating effects due to activity through the active stress tensor term $\boldsymbol{\sigma }_\textrm{a}$ first proposed by Simha and Ramaswamy [1],

1 $$\begin{aligned} \boldsymbol{\sigma }_\textrm{a} = \zeta (\textbf{n} \otimes \textbf{n}), \end{aligned}$$

where $\textbf{n}$ is the nematic director (the average orientation of the constituent agents) and the outer product is defined by $(\textbf{n} \otimes \textbf{n})_{ij} = n_{i} n_{j}$. This form of active stress means that distortion of the director will generate a flow, as has been observed in many publications [2, 7, 10, 57]. The coefficient $\zeta$ is the activity parameter, which can be positive or negative, and has the dimensions of pressure. The magnitude of $\zeta$ quantifies the stress the active agents exert on their surroundings, and the direction of the generated flow is determined by the sign of $\zeta$, with agents either pushing the fluid out or pulling the fluid in along their long axis. This simple description of “pushers” and “pullers” to describe active agents is commonly replaced by the terms “extensile” and “contractile”, respectively. A schematic illustration of these two contrasting behaviours for active agents is shown in Fig. 1, where $\zeta <0$ for extensile agents and $\zeta >0$ for contractile agents [9, 42, 43, 57]. Note that the opposite sign of the activity parameter in (1) is sometimes used, for instance, in [7, 8, 10, 81]. The presence of active stress leads to a transition to a flowing state with an activity parameter of magnitude greater than a critical value. For an initially planar state (as described in this paper, albeit with a small pre-tilt at the bounding surfaces), the instability to a flowing state occurs for extensile systems. However, if an initial homeoptropic alignment was considered, the instability of the active nematic would occur for contractile systems. In all cases, for the zero-field case the fastest growing mode of instability exhibits a director distortion that is antisymmetric about the middle of the layer (see, for instance, [8]).

The aim of this article is to model mathematically the hydrodynamic response of an active nematic liquid crystal to an external orienting field. We solve the Ericksen–Leslie equations numerically, firstly considering the dynamic response of the director orientation and flow when an external orienting field is switched on, and then examine the steady-state solutions when both the activity and orienting field are changed simultaneously. We then take particular note of the dynamic response when an orienting field is switched off, focussing on how the activity strength can either enhance or reduce kickback in active nematics and generate nontrivial steady-state solutions.

**Fig. 2:**
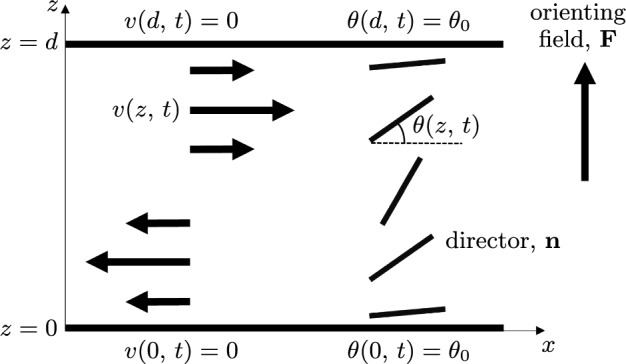
An active nematic liquid crystal occupying a channel between two solid boundaries at $z=0$ and $z=d$, subject to an external orienting field $\textbf{F}$. The nematic director $\textbf{n}$, indicated by the thick black lines, lies in the $(x,\,z)$-plane, so it is characterised by the director tilt angle $\theta (z,\,t)$. The director is infinitely anchored at nonzero pre-tilt angle $\theta _0$ at both channel boundaries. The fluid velocity $v(z,\,t)$, indicated by the black arrows, is assumed to be parallel to the *x*-direction and satisfies a no-slip condition on the boundaries

## Mathematical model

We consider a channel of active nematic liquid crystal, confined between two parallel solid boundaries at $z=0$ and $z=d$, and subject to an external orienting field, $\textbf{F}$, parallel to the *z*-direction (see Fig. 2). The nematic director $\textbf{n}$ is assumed to lie in the $(x,\,z)$-plane and is expressed in the form $\textbf{n}= \bigl (\cos \theta (z,\,t),\, 0,\, \sin \theta (z,\,t)\bigr )$, where $\theta (z,\,t)$ is the director tilt angle measured with respect to the *x*-direction. Through surface interactions, which may be induced via a surface treatment, the director is assumed to be infinitely anchored at a fixed pre-tilt angle $\theta _0$ such that $\theta (0,\, t)= \theta (d,\,t)=\theta _0$. Such a director pre-tilt is often imposed on the boundaries for inactive nematics systems to break the symmetry and reduce defects by ensuring that the orienting field creates a homogeneous director distortion throughout the *xy*-plane of the device. The experimental technique used to align the director at the same pre-tilt angle along both channel boundaries is known as anti-parallel rubbed alignment and is induced through physical rubbing of the two boundaries in opposite directions. The alternative scenario, which will not be considered in this article, where the rubbing directions along the channel boundaries are the same is termed parallel rubbed alignment and leads to director pre-tilt angles which are equal in magnitude but opposite in sign. It has previously been shown that when nematic cells are constructed using parallel rubbing and subject to a sufficiently high applied voltage (i.e. above the Freedericksz transition threshold), the director does not exhibit any kickback following the removal of the voltage − instead, the flow enhances director relaxation during switch-off through a reduced effective nematic viscosity [71, 79]. The fluid velocity, *v*(*z*, *t*), is constrained to be parallel to the *x*-direction due to the presence of solid channel boundaries, translational invariance in the *x*-direction and director rotation only occurring in the *xz*-plane, and satisfies a no-slip condition $v(0,t) = v(d,t) = 0$ for all time. When the timescale due to changes in director orientation is significantly larger than that for the velocity, it is common to make the approximation that fluid inertia is negligible [79, 80], which we assume to be the case here. We also make the assumption that there is no applied pressure gradient, so that our present situation leads to a spatially independent effective pressure throughout the system.

An external orienting field $\textbf{F}$, which we assume to be spatially uniform and time-independent, is applied perpendicular to the boundaries of the channel such that $\textbf{F} = (0, 0, F)$. If we were to interpret this orienting field as an electric or magnetic field, then the appropriate Maxwell equations would also need to be satisfied [77, 79]. We account for changes in the director orientation due to an orienting field by including a field energy density, $w_{\textrm{field}} = - \frac{1}{2} \chi _{f} (\textbf{n} \cdot \textbf{F})^2$, which is of similar form to that of an electric or magnetic energy density used when modelling inactive nematics. The coefficient $\chi _{f}$ is defined as the anisotropic susceptibility of the active nematic to reorient in the field. We assume throughout this investigation that $\chi _{f}$ is positive, which means that the energetic preference is for the director to align parallel to the applied field. In inactive nematics, the cases of applied magnetic and electric fields lead to a critical threshold value of *F* (the Freedericksz threshold), below which the director remains aligned with the boundary orientation and above which the director orientation increases to a maximum angle approaching $\theta \approx \pi /2$ radians in the bulk of the channel. The latter state, the *switched state*, includes reorientational boundary layers close to the boundaries, within which the director angle reorients from the bulk value of $\pi /2$ to the infinite anchoring at the pre-tilt angle boundary condition $\theta _0$ (see [77, 79]).

The dynamics of the director angle and fluid velocity governed by the Ericksen–Leslie equations [31–33], which include the activity stress tensor (1) and the possibility of an applied orienting field,

2 $$\begin{aligned} \gamma _{1} \theta _{t}&= f(\theta ) \theta _{zz} + \frac{1}{2} f'(\theta ) (\theta _{z})^2\nonumber \\&\quad + \chi _{f} F^2 \sin \theta \cos \theta - m(\theta ) v_{z}, \end{aligned}$$

3 $$\begin{aligned} 0&=\big (g(\theta ) v_{z} + m(\theta ) \theta _{t} + \zeta \cos \theta \sin \theta \big )_{z}, \end{aligned}$$

where the elasticity function $f(\theta )$ and viscosity functions $m(\theta )$, $g(\theta )$ are defined by

4 $$\begin{aligned} f(\theta )&= K_{1} \cos ^2 \theta + K_{3} \sin ^2 \theta , \end{aligned}$$

5 $$\begin{aligned} m(\theta )&=\frac{1}{2}\big (\gamma _{1} + (\eta _{1}-\eta _{2}) \cos (2 \theta ) \big ), \end{aligned}$$

6 $$\begin{aligned} g(\theta )&= \eta _{1} \cos ^2 \theta + \eta _{2} \sin ^2 \theta + \eta _{12} \sin ^2 \theta \cos ^2 \theta . \end{aligned}$$

In the director angle Eq. (2) and flow Eq. (3), $K_{1}$ and $K_{3}$ are the Frank elastic constants associated with, respectively, splay and bend director distortions, $\zeta$ is the activity parameter, $\gamma _{1}$ is the rotational viscosity, while $\eta _{1},\,\eta _{2},\,\eta _{12}$ are Miesowicz viscosities. The product $\chi _{f}F^2$ is termed the orienting field coefficient. The subscripts *t* and *z* denote the partial derivative with respect to that independent variable. The elasticity function in Eq. (4) is the effective elastic constant of the liquid crystal, which changes depending on the director orientation. The viscosity function in Eq. (5) models the coupling between the rotation of the director, $\theta _{t}$, and the shear rate, $v_{z}$, and the effective viscosity of the liquid crystal is determined from the definition in Eq. (6) and changes depending on the director orientation.

We will solve Eqs. (2) and (3) subject to the boundary conditions for the director angle and flow velocity

7 $$\begin{aligned}&\theta (0,\,t) =\theta _0, \quad v(0,\,t)= 0, \end{aligned}$$

8 $$\begin{aligned}&\theta (d,\,t) =\theta _0, \quad v(d,\,t) = 0, \end{aligned}$$

with an initial condition for the director of

9 $$\begin{aligned} \theta (z, \, 0) = \theta _0. \end{aligned}$$

Since we have neglected the inertia term in (3), we do not have the freedom to prescribe an initial condition for the flow velocity. Instead, the initial value *v*(*z*, 0) is obtained from (2), (3), using the substitution $\theta \equiv \theta _0$.

In the calculations that follow, we use the material parameters measured for the nematic liquid crystal 5CB [77] and a channel width of $d = 10\,\upmu$m. Furthermore, a small director pre-tilt on both boundaries is assumed, $\theta _0= 0.01$ rad. This mirrors the material and geometric parameters that were previously considered in [79]. As discussed in [57], the material parameters for many active nematics are yet to be fully characterised. Despite the difference in length scales between the constituent agents in inactive nematics (nanometre scale) and in active fluids (micrometre scale), as well as the lack of experimental data on active nematic material parameters, we propose that the behaviour in this report using inactive nematic parameters will be qualitatively similar for an active nematic. In order to examine how the activity of the liquid crystal influences kickback when an orienting field is switched off, we consider orienting field strengths $\chi _{f}F^2 = 10{-}100$ Pa, all of which are above the equivalent Freedericksz transition in inactive nematics. To analyse the hydrodynamic behaviour of the active nematic liquid crystal, we will consider the director angle at the channel centre, $\theta _{{d/2}}(t)=\theta (d/2,\, t)$, and the flow velocity at the quarter point in the channel, $v_{{d/4}}(t)=v(d/4,\, t)$. We will consider not only how these measures change with time, but also their steady-state values (i.e. in the limit as *t* becomes large).

In this article, we consider the lowest-order mode, i.e. the mode with the least director distortion and the largest wavelength. The results for extensile agents and contractile agents will both be considered. Higher-order mode solutions will not be considered here as they involve large elastic distortions of the director and, as has previously been found, are often either unstable or metastable [57]. We obtain numerical solutions of the system (2)–(9) using the finite-element package COMSOL Multiphysics [82].

## Switch on: dynamics and steady-state solutions

**Fig. 3:**
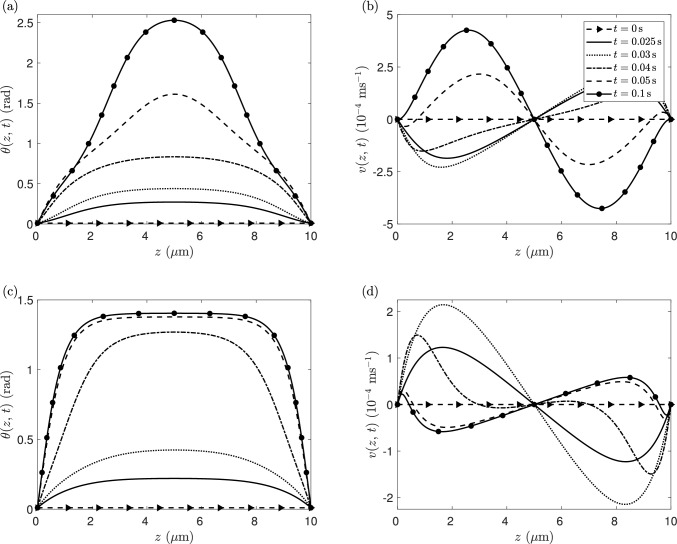
Evolution of the director angle $\theta (z,\,t)$ and the velocity $v(z,\,t)$ with time when an orienting field of strength $\chi _{f}F^2=10$ Pa is applied. The profiles **a**, **b** are for the extensile system when $\zeta =-25\,$Pa, while **c**, **d** correspond to the contractile system for $\zeta =25\,$Pa

The dynamics and steady-state solutions for the director orientation and flow velocity when an external orienting field is switched on are now considered for both extensile and contractile systems. Typical director angle and flow velocity evolutions are shown in Fig. 3, where we have used an orienting field strength of $\chi _{f}F^2=10$ Pa and activity parameter values of $\zeta =-25\,$Pa (extensile) and $\zeta =25\,$Pa (contractile). For comparison, the critical Freedericksz transition in 5CB for this cell thickness would be $\chi _{f}F^2=0.61$ Pa and the zero-field critical activity parameter would be $\zeta =-13.87\,$Pa. For both extensile and contractile agents, the director orientation increases from an initial uniform configuration of $\theta _0=0.01$ rad across the channel, to a switched director distortion in the bulk which, in contrast to such a system without an applied orienting field, is now symmetric about the middle of the layer.

In the extensile case, Fig. 3a shows that the activity of the liquid crystal and the orienting field combine to generate a steady-state director orientation with $\theta > \pi /2$ in most of the channel, with large director gradients occurring either side of the channel centre. The regions of positive and negative director gradient are coupled with steady-state positive and negative flow, with the maximum and minimum flow occurring close to the quarter points in the cell, *v*(*d*/4, *t*) and *v*(3*d*/4, *t*), respectively. However, in Fig. 3b we see that the evolution to steady state occurs through a period of backflow, in other words, when the flow is temporarily in the opposite direction to the eventual steady-state flow. In the contractile case, a situation in which an undistorted director angle and zero flow would be the stable state without an orienting field [57], Fig. 3c shows that the application of the field leads to a steady-state director orientation for which $0<\theta < \pi /2$ throughout the channel and is almost uniform in the bulk, away from the reorientation layers close to the channel boundaries. As in the extensile case, regions of positive and negative director gradient correspond to positive and negative flow, although these are now restricted to regions very close to the boundaries (see Fig. 3d). The central region of uniform director orientation at steady state coincides with an almost constant positive shear, $v_{z} > 0$. Again we see that the evolution to steady state occurs through a period of backflow in which the flow is in the opposite direction to the steady state.

**Fig. 4:**
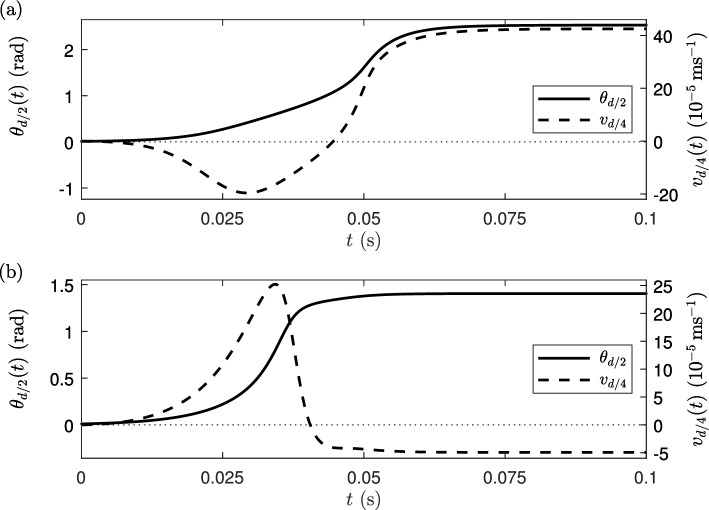
Evolution of $$\theta _{{d/2}}(t)$$ and $$ v_{{d/4}}(t)$$, for **a** the extensile system when activity $$\zeta =-25$$ Pa and **b** the contractile system when activity $$\zeta =25$$ Pa, and for an orienting field $$\chi _{f}F^2=10$$ Pa is switched on

To illustrate the effect of backflow on the dynamics of the extensile system as it approaches switch-on steady state, Fig. 4a shows how the director angle and flow velocity for the extensile system change with time after the orienting field is switched on at $t=0$. There is a initial slow increase in the director angle which coincides with a negative backflow (i.e. $v < 0$) which reaches a maximum magnitude at around time $t = 0.03$ s. Subsequently, the backflow reduces, the flow direction reverses and the director angle increases rapidly towards its steady-state alignment. As also observed in Fig. 3a, b, by time $t=0.1$ s the system has reached a switched-on steady-state state where both the flow at $z=d/4$ and the director orientation at $z=d/2$ exhibit their maximum values. In Fig. 4b we see that, for a contractile system, the system also exhibits activity-induced backflow; in this case, a positive flow velocity becomes increasingly pronounced, reaching a maximum at $t \approx 0.035$ s. After this time, the flow decreases and eventually reverses direction and the director orientation in the centre of the channel increases towards its steady state below $\theta =\pi /2$.

**Fig. 5:**
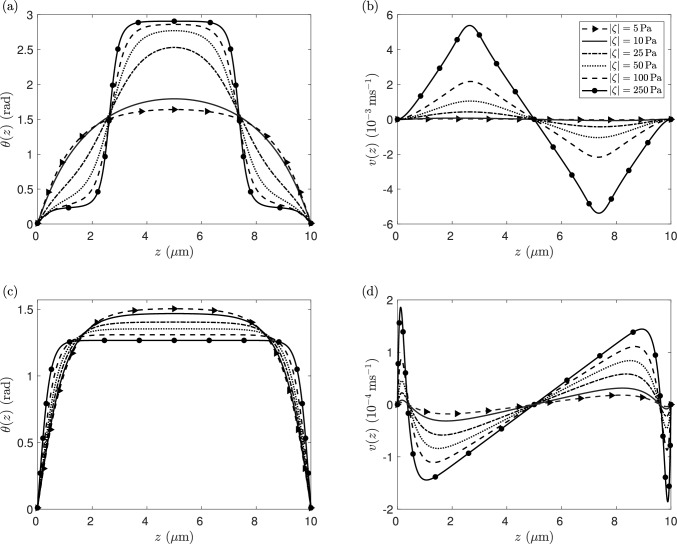
Steady-state solutions for the director angle $\theta (z)$ and the velocity *v*(*z*) at different activity magnitudes when an orienting field of strength $\chi _{f}F^2=10$ Pa is applied. The profiles **a**, **b** are for the extensile system ($\zeta < 0)$, while **c**, **d** correspond to the contractile system ($\zeta > 0)$

In Fig. 5, we plot the steady-state solutions, for both extensive and contractile systems at different levels of activity magnitude, for an orienting field $\chi _{f}F^2 = 10$ Pa. For the extensile system (Fig. 5a, b with $\zeta <0$), increasing the activity magnitude leads to the formation of regions of uniform director orientation at the channel centre and close to the boundaries. These regions are connected by internal reorientation layers that become increasingly sharp as the activity magnitude increases. As discussed in [57], in the asymptotic limit of $\zeta \rightarrow - \infty$, the preferred director orientation is $\theta = n \pi \pm \theta _\textrm{L} \,\ (n \in \mathbb {Z})$, where the Leslie angle, $\theta _\textrm{L}$, also termed the flow alignment angle [77], is the preferred director tilt in simple rectilinear shear for flow-aligning nematics and is defined by

10 $$\begin{aligned} \theta _\textrm{L} = \tan ^{-1} \left( \sqrt{\frac{\eta _{1}-\eta _{2}+\gamma _1}{\eta _{1}-\eta _{2}-\gamma _1} } \right) . \end{aligned}$$

Using the material parameters for 5CB, $\theta _\textrm{L} \approx 0.208$ rad, with a corresponding Leslie viscosity $\eta _\textrm{L}= g( \theta _\textrm{L}) \approx 23.8$ mPa s. From Fig. 5a, there are three regions of uniform flow alignment in the limit $\zeta \rightarrow - \infty$, one close to the channel centre where the director angle exhibits flow alignment at the “negative” Leslie angle $\theta = \pi - \theta _\textrm{L}$, and two close to the channel boundaries at the “positive” Leslie angle $\theta = \theta _\textrm{L}$. As is standard for inactive nematics [83, 84], the regions of negative and positive Leslie angle correspond to, respectively, negative and positive velocity gradients, with the active agents moving at a faster speed throughout most of the channel as $\zeta \rightarrow - \infty$.

**Fig. 6:**
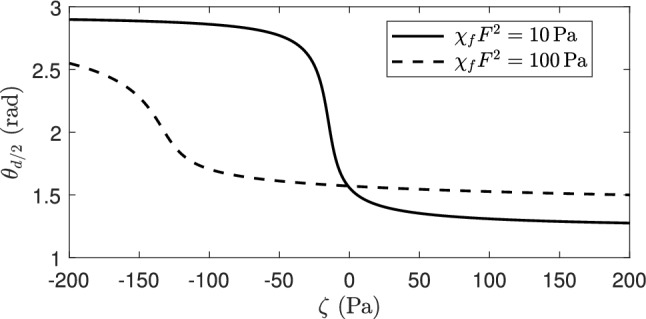
Variation of the director angles $\theta _{{d/2}}$ with the activity parameter $\zeta$ at orienting fields $\chi _{f} F^2 = 10$ and 100 Pa. The application of large orienting fields leads to continuous, steady-state solution branches which exist for all activities

For the contractile system (Fig. 5c, d where $\zeta >0$), increasing the activity parameter leads to a reduction in the director orientation in most of the channel, with a uniform director orientation in the bulk of the channel and reorientation layers close to the boundaries. The analysis in Walton et al. [57] demonstrated that, in the absence of an orienting field, the boundary layers in the director profile behave like $|\theta _{z}| \sim \sqrt{\zeta }$ as $\zeta \rightarrow \infty$, with bulk orientation $\theta = \theta ^{*} \approx 1.199$ rad and an effective viscosity $g^*=g(\theta ^*)\approx 93.3$ mPa s for 5CB material parameter values. The flow is characterised by localised jets which increase in magnitude and become increasingly sharp as activity increases, with significant areas of positive velocity gradient where the director aligns at its activity-induced orientation $\theta ^{*}$. This behaviour is again seen here, as would be expected when the activity increases and dominates the effects of an orienting field. The key difference in the present system compared to a system without an aligning field is that the transition between the nontrivial solution for an extensile system and the nontrivial solution for a contractile system is continuous, with the director and flow profiles deforming continuously as activity varies. This can be seen clearly in Fig. 6 and is in contrast to the discontinuous transition found in the no-field situation [57].

The formation of a solution branch which allows for a continuous transition between nontrivial solutions for extensile and contractile systems bears some resemblance to the behaviour exhibited by an active nematic subject to an external pressure gradient in the *x*-direction [57]. An important difference between the field-driven and pressure-driven systems is that the solutions for the director orientation in [57] are asymmetric as a result of the Poiseuille flow induced by the pressure gradient, whereas they remain symmetric when a large orienting field is applied with anti-parallel alignment boundary conditions for the director.

## Switch off: kickback in active nematics

We now consider the behaviour of the system when, having reached steady state with an applied orienting field, the field is then switched off, i.e. the field strength parameter $\chi _{f}F^2$ is set to 0 Pa in Eq. (2). If we use $\theta _\textrm{on}^\infty (z)$ and $v_\textrm{on}^\infty (z)$ to denote, respectively, the steady-state director orientation and flow velocity immediately prior to switch-off for a nonzero orienting field, we solve the switch-off Ericksen–Leslie equations numerically, subject to the boundary conditions (7) and (8), and “initial” conditions

11 $$\begin{aligned} \theta (z, \, t_\textrm{off}) = \theta _\textrm{on}^\infty (z),\quad v(z,\, t_\textrm{off})=v_\textrm{on}^\infty (z). \end{aligned}$$

**Fig. 7:**
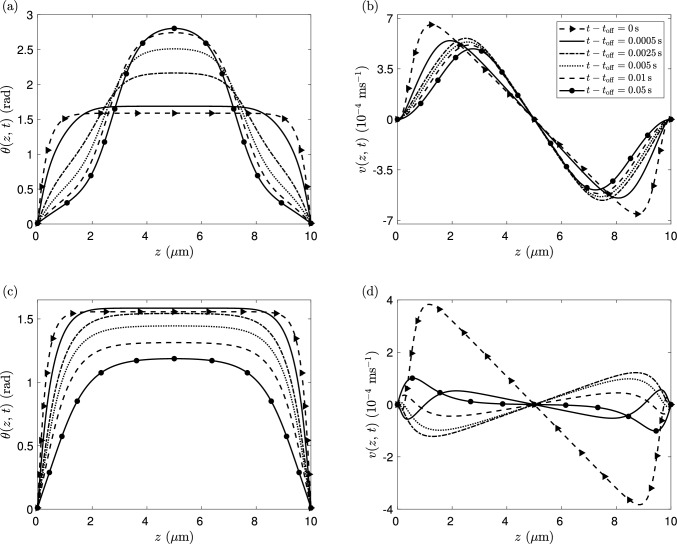
Director angle $\theta (z,\,t)$ and the velocity $v(z,\,t)$ when an orienting field of strength $\chi _{f}F^2=100$ Pa is switched off. The profiles **a**, **b** are for the extensile system when $\zeta =-25\,$Pa, while **c**, **d** correspond to the contractile system for $\zeta =25\,$Pa

Figure 7 shows the response of the director angle and flow velocity for $|\zeta |=25$ Pa shortly after an orienting field of strength $\chi _{f}F^2=100$ Pa is switched off. Figure 7a, c shows that the director orientation at the channel centre initially increases for both agents. However, for the extensile system, we see a monotonic change in director angle at every location in the region until the equilibrium value is attained, whereas in the contractile system the director in the centre of the region initially increases and then later decreases. This nonmonotonic behaviour is equivalent to the kickback effect seen in inactive nematics when a high magnitude orienting field is removed. In the contractile system this kickback effect coincides with a reversal in the flow velocity, as seen in Fig. 7d immediately after switch-off. Neither the kickback effect nor the change in the flow direction is observed for the extensile system in Fig. 7a, b.

**Fig. 8:**
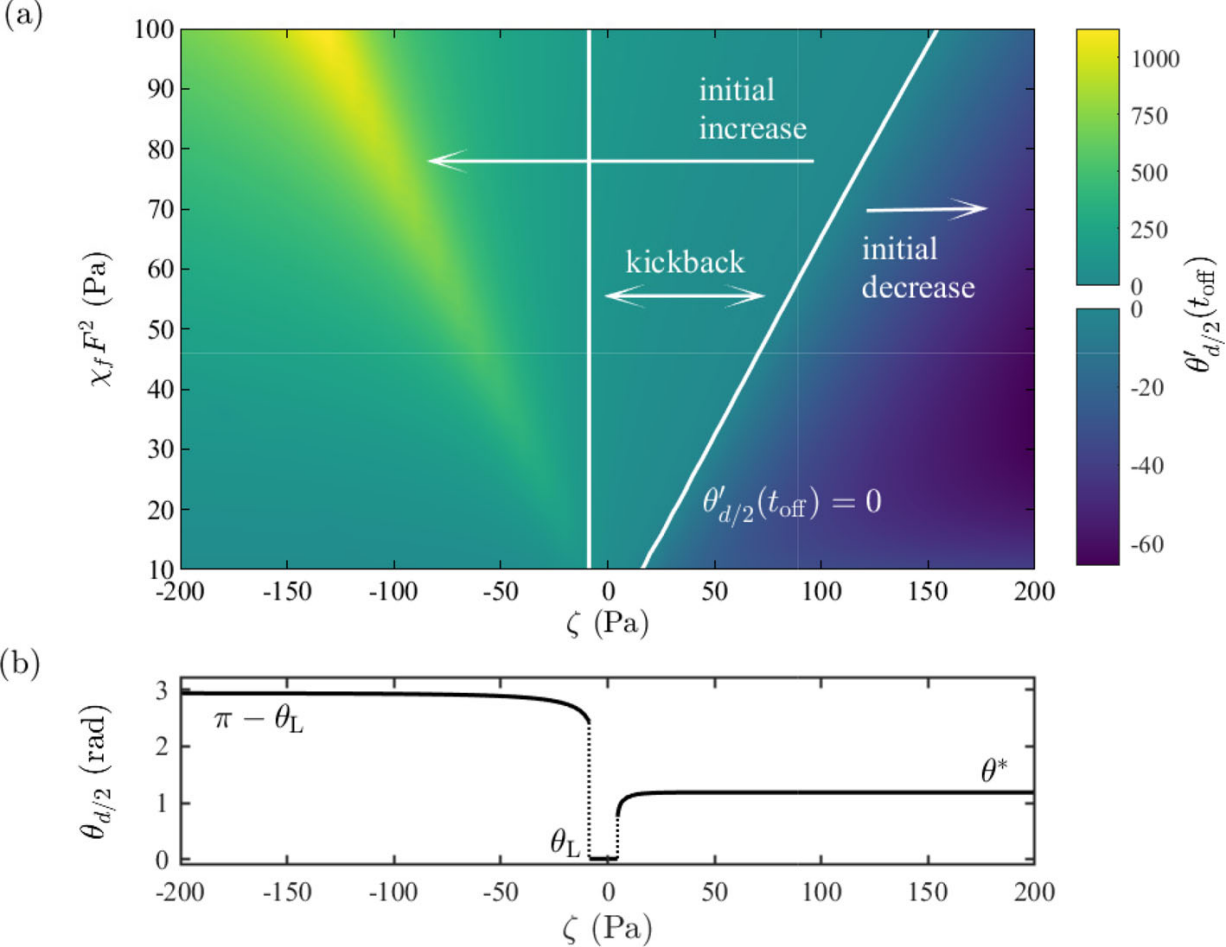
**a** Kickback in activity/orienting field space, $(\zeta , \chi _{f}F^2)$. The coloured background indicates the value of $\theta _{{d/2}}'(t_{\textrm{off}})$, i.e. immediately after the orienting field is removed. Kickback occurs between the two white lines, the region where $\theta _{{d/2}}'(t_{\textrm{off}})>0$ and $\theta _{{d/2}}'(t)=0$ for some time $t>t_{\textrm{off}}$. **b** The long-time director tilt angle in the centre of the channel, as the system reaches the steady switched-off state

The presence of nonmonotonic director behaviour in the centre of the region for a range of activities and field strengths is shown in Fig. 8a, where the coloured background denotes the initial rotation of the director, $\theta _{{d/2}}'(t_{\textrm{off}})$, and the right line indicates where $\theta _{{d/2}}'(t_{\textrm{off}})=0$. For activities and field strengths to the left of this line the director angle initially increases, while to the right the director angle initially reduces. Figure 8b plots the steady-state director angle in the centre of the channel after the field is switched off. As discussed in Walton et al. [57] and in Sect. 3, the no-field steady-state director profiles for extensile systems correspond to $\theta _{d/2}\approx \pi -\theta _{\textrm{L}}$, above the field-on profile where $\theta _{d/2}$ is close to $\pi /2$ radians. Therefore, the persistent director angle increase for $\zeta <0$ in Fig. 8a is to be expected. For contractile agents, the director aligns at $\theta _{d/2}\approx \theta ^{\mathrm {*}}$ once the field is removed, below $\pi /2$ radians. The only exception is a small interval $\zeta \in (-8.6,4.6)$ where the director in the bulk of the channel aligns at the much smaller Leslie angle $\theta _{\textrm{L}}$. The vertical line in Fig. 8a corresponds to the left end point of the narrow interval. To the left of the line $\zeta =-8.6$ Pa, the director angle increases monotonically for all time. In this situation, the effect of activity-induced flow is to accelerate the motion towards the steady state, as would occur in an inactive nematic with parallel rubbed anchoring. Kickback only occurs in the region between the two lines in Fig. 8a, with the director angle initially increasing before reaching a maximum and then decreasing to the switched-off steady-state profile. For any particular value of the orienting field strength, a sufficiently contractile ($\zeta >0$) system will lead to the removal of the kickback effect. However, it is notable that for contractile systems, the fastest acceleration to the stable state occurs at high activity but for intermediate orienting field strengths (the darkest area at the right of Fig. 8a). The removal of kickback in sufficiently contractile systems can be attributed to the active flow profile deriving from a symmetric director distortion. In Fig. 7d we see that the flow reverses as the contractile system relaxes (see time $t=0.0025\,\textrm{s}$ versus $t=0\,\textrm{s}$), whereas in the extensile system the flow has no reversal and, instead, persists as the director profile relaxes. Consequently, the extensile active flow driven by the symmetric director profile leads to an increase of the director in the middle of the layer. Conversely, the contractile active flow driven by the symmetric director profile tends to negate the classical backflow and thus, for sufficiently contractile systems, remove kickback altogether.

## Conclusions

In this article, we have examined the effects of an external orienting field on the orientation and flow of an active nematic liquid crystal in a channel using an adapted version of the Ericksen–Leslie equations. Our model shows that the application of the orienting field leads to the formation of a solution branch which allows for a continuous transition between nontrivial contractile and nontrivial extensile steady-state solutions. These solutions were previously shown to be stable and disconnected from each other in the absence of any external forcing (e.g. a pressure gradient or orienting field). The formation of a single continuous branch due to an external force which exists for all activity values has been observed in a previous investigation into pressure-driven flow of an active nematic within a channel, except that for the present case of an orienting field there is no break in symmetry of the director structure. We have, therefore, discovered another mechanism which can be used in experiments to promote the existence of nontrivial solutions for an active nematic within a channel, with the solutions maintaining their symmetry. The dynamic response of extensile and contractile agents when the orienting field is switched off was then considered. For extensile agents, a monotonic increase in the director orientation at the channel centre was observed until the equilibrium state was reached. The flow direction did not change immediately upon removal the field, and so the initial increase in director angle at the channel centre is not classified as kickback. Instead, the flow enhances the rotation of the director towards its equilibrium configuration. The dynamic response of contractile agents after switch-off demonstrated that either nonmonotonic behaviour (an initial increase followed by a reduction towards equilibrium in this case), or a monotonic decrease towards equilibrium was possible, depending on the combination of the switch-on field and activity values. The nonmonotonic behaviour in the director orientation at the channel centre demonstrates that contractile active nematics may undergo kickback. Our numerical calculations show that kickback can be made less pronounced, and eventually completely avoided, for contractile agents with high level of positive activity, even when an orienting field in excess of the corresponding Freedericksz transition is removed. This removal of kickback is due to the contractile active flow caused by a symmetric director distortion negating the classical backflow. Given the level of interest in the control and design of active nematic liquid crystals, and their applications, we would hope that our work will aid future research into using orienting fields to optimise the design of active fluid systems.

## Data Availability

All methods to produce the data of the paper are available within the paper itself.

## References

[1] R.A. Simha, S. Ramaswamy, Phys. Rev. Lett. **89**, 058101 (2002). 10.1103/PhysRevLett.89.058101 10.1103/PhysRevLett.89.058101 12144468

[2] Y. Hatwalne, S. Ramaswamy, M. Rao, R.A. Simha, Phys. Rev. Lett. **92**, 118101 (2004). 10.1103/physrevlett.92.118101 15089176 10.1103/PhysRevLett.92.118101

[3] M.C. Marchetti, J.F. Joanny, S. Ramaswamy, T.B. Liverpool, J. Prost, M. Rao, R.A. Simha, Rev. Mod. Phys. **85**, 1143 (2013). 10.1103/revmodphys.85.1143

[4] W. Yan, J.F. Brady, New J. Phys. **20**, 053056 (2018). 10.1088/1367-2630/aac3c5

[5] Z. Lin, S. Chen, T. Gao, J. Fluid Mech. **921**, 25 (2021). 10.1017/jfm.2021.531

[6] D. Cortese, J. Eggers, T.B. Liverpool, EPL **115**, 28002 (2016). 10.1209/0295-5075/115/28002

[7] S.P. Thampi, J.M. Yeomans, Eur. Phys. J. Spec. Top. **225**, 651 (2016). 10.1140/epjst/e2015-50324-3

[8] R. Voituriez, J.F. Joanny, J. Prost, EPL **70**, 404 (2005). 10.1209/epl/i2004-10501-2

[9] L. Giomi, M.C. Marchetti, T.B. Liverpool, Phys. Rev. Lett. **101**, 198101 (2008). 10.1103/physrevlett.101.198101 19113315 10.1103/PhysRevLett.101.198101

[10] S.A. Edwards, J.M. Yeomans, EPL **85**, 18008 (2009). 10.1209/0295-5075/85/18008

[11] F. Bonelli, G. Gonnella, A. Tiribocchi, D. Marenduzzo, Eur. Phys. J. E **39**, 1 (2016). 10.1140/epje/i2016-16001-2 26769011 10.1140/epje/i2016-16001-2

[12] V.J. Pratley, E. Caf, M. Ravnik, G.P. Alexander, Commun. Phys. **7**, 127 (2024). 10.1038/s42005-024-01611-y

[13] A.J.H. Houston, N.J. Mottram, Commun. Phys. **7**, 375 (2024). 10.1038/s42005-024-01864-7 39574428 10.1038/s42005-024-01864-7 PMC11576538

[14] H.R. Brand, H. Pleiner, D. Svenšek, Eur. Phys. J. E **37**, 83 (2014). 10.1140/epje/i2014-14122-2

[15] H.R. Brand, H. Pleiner, D. Svenšek, Rheol. Acta **57**, 773 (2018). 10.1007/s00397-018-1112-x

[16] D. Marenduzzo, E. Orlandini, M.E. Cates, J.M. Yeomans, Phys. Rev. E **76**, 031921 (2007). 10.1103/physreve.76.031921 10.1103/PhysRevE.76.031921 17930285

[17] S.P. Thampi, R. Golestanian, J.M. Yeomans, Phil. Trans. R. Soc. A **372**, 20130366 (2013). 10.1098/rsta.2013.0366

[18] L. Giomi, M.J. Bowick, X. Ma, M.C. Marchetti, Phys. Rev. Lett. **110**, 228101 (2013). 10.1103/physrevlett.110.228101 23767749 10.1103/PhysRevLett.110.228101

[19] L. Giomi, M.J. Bowick, P. Mishra, R. Sknepnek, M.C. Marchetti, Phil. Trans. R. Soc. A **372**, 20130365 (2014). 10.1098/rsta.2013.0365 25332389 10.1098/rsta.2013.0365 PMC4223672

[20] E. Putzig, G.S. Redner, A. Baskaran, A. Baskaran, Soft Matter **12**, 3854 (2016). 10.1039/c6sm00268d 26983376 10.1039/c6sm00268d PMC5166704

[21] D. Cortese, J. Eggers, T.B. Liverpool, Phys. Rev. E **97**, 022704 (2018). 10.1103/physreve.97.022704 29548179 10.1103/PhysRevE.97.022704

[22] C.A. Whitfield, R.J. Hawkins, New J. Phys. **18**, 123016 (2016). 10.1088/1367-2630/18/12/123016

[23] S. Chandragiri, A. Doostmohammadi, J.M. Yeomans, S. Thampi, Phys. Rev. Lett. **125**, 148002 (2020). 10.1103/physrevlett.125.148002 33064508 10.1103/PhysRevLett.125.148002

[24] M.R. Nejad, J.M. Yeomans, Phys. Rev. Lett. **128**, 048001 (2022). 10.1103/physrevlett.128.048001 35148135 10.1103/PhysRevLett.128.048001

[25] R. Alert, J. Phys. A: Math. Theor. **55**, 234009 (2022). 10.1088/1751-8121/ac6c61

[26] R. Assante, D. Corbett, D. Marenduzzo, A. Morozov, Soft Matter **19**, 189 (2023). 10.1039/d2sm01188c 36503973 10.1039/d2sm01188c

[27] A.J.H. Houston, G.P. Alexander, Front. Phys. **11**, 1110244 (2023). 10.3389/fphy.2023.1110244

[28] D. Svenšek, H. Pleiner, H.R. Brand, Soft Matter **15**, 2032 (2019). 10.1039/C9SM00023B 30724307 10.1039/c9sm00023b

[29] A. Doostmohammadi, J. Ignés-Mullol, J.M. Yeomans, F. Sagués, Nat. Commun. **9**, 3246 (2018). 10.1038/s41467-018-05666-8 30131558 10.1038/s41467-018-05666-8 PMC6104062

[30] J.L. Ericksen, Trans. Soc. Rheol. **5**, 23 (1961). 10.1122/1.548883

[31] F.M. Leslie, Q., J. Mech. Appl. Math. **19**, 357 (1966). 10.1093/qjmam/19.3.357

[32] F.M. Leslie, Arch. Rat. Mech. Anal. **19**, 265 (1968). 10.1007/bf00251810

[33] F.M. Leslie, Contin. Mech. Thermodyn. **4**, 167 (1992). 10.1007/bf01130288

[34] D. Banerjee, A. Souslov, A.G. Abanov, V. Vitelli, Nat. Commun. **8**, 1573 (2017). 10.1038/s41467-017-01378-7 29146894 10.1038/s41467-017-01378-7 PMC5691086

[35] C.A. Whitfield, T.C. Adhyapak, A. Tiribocchi, G.P. Alexander, D. Marenduzzo, S. Ramaswamy, Eur. Phys. J. E **40**, 50 (2017). 10.1140/epje/i2017-11536-2 28429181 10.1140/epje/i2017-11536-2

[36] G. Duclos, C. Blanch-Mercader, V. Yashunsky, G. Salbreux, J.F. Joanny, J. Prost, P. Silberzan, Nat. Phys. **15**, 868 (2019). 10.1038/s41567-019-0544-2 31467586 10.1038/s41567-019-0544-2 PMC6715445

[37] D. Banerjee, A. Souslov, V. Vitelli, Phys. Rev. Fluids **7**, 043301 (2020). 10.1103/physrevfluids.7.043301

[38] L.A. Hoffmann, K. Schakenraad, R.M.H. Merks, L. Giomi, Soft Matter **16**, 764 (2020). 10.1039/c9sm01851d 31830190 10.1039/c9sm01851d

[39] B. Zhang, B. Hilton, C. Short, A. Souslov, A. Snezhko, Phys. Rev. Res. **2**, 043225 (2020). 10.1103/physrevresearch.2.043225

[40] B. Zhang, A. Snezhko, Phys. Rev. Lett. **128**, 218002 (2022). 10.1103/physrevlett.128.218002 35687470 10.1103/PhysRevLett.128.218002

[41] L. Giomi, M.C. Marchetti, Soft Matter **8**, 129 (2012). 10.1039/c1sm06077e

[42] X.-G. Yang, M.G. Forest, Q. Wang, Chin. Phys. B **23**, 118701 (2014). 10.1088/1674-1056/23/11/118701

[43] X.-G. Yang, Q. Wang, Soft Matter **12**, 1262 (2016). 10.1039/c5sm02115d 26583506 10.1039/c5sm02115d

[44] L. Chen, C.-F. Lee, J. Toner, New J. Phys. **20**, 113035 (2018). 10.1088/1367-2630/aaec31

[45] B. Zhang, H. Yuan, A. Sokolov, M. Olvera de la Cruz, A. Snezhko, Nat. Phys. **18**, 154 (2022). 10.1038/s41567-021-01442-6

[46] A. Amiri, R. Mueller, A. Doostmohammadi, J. Phys. A: Math. Theor. **55**, 094002 (2022). 10.1088/1751-8121/ac4abe

[47] A. Singh, Q. Vagne, F. Jülicher, I.F. Sbalzarini, Phys. Rev. Res. **5**, L022061 (2023). 10.1103/physrevresearch.5.l022061

[48] J. Hardoüin, R. Hughes, A. Doostmohammadi, J. Laurent, T. Lopez-Leon, J.M. Yeomans, J. Ignés-Mullol, F. Sagués, Commun. Phys. **2**, 121 (2019). 10.1038/s42005-019-0221-x

[49] A. Opathalage, M.M. Norton, M.P.N. Juniper, B. Langeslay, S.A. Aghvami, S. Fraden, Z. Dogic, PNAS **116**, 11 (2019). 10.1073/pnas.1816733116 30804207 10.1073/pnas.1816733116 PMC6421422

[50] A. Samui, J.M. Yeomans, S.P. Thampi, Soft Matter **17**, 10640 (2021). 10.1039/d1sm01434j 34788355 10.1039/d1sm01434j

[51] D.A. Khaladj, L.S. Hirst, Front. Phys. **10**, 880941 (2022). 10.3389/fphy.2022.880941

[52] W. Li, L. Li, Q. Shi, M. Yang, N. Zheng, Soft Matter **18**, 5459 (2022). 10.1039/D2SM00134A 35822840 10.1039/d2sm00134a

[53] S. Alam, B. Najma, A. Singh, J. Laprade, G. Gajeshwar, H.G. Yevick, A. Baskaran, P.J. Foster, G. Duclos, Phys. Rev. X **14**, 041002 (2024). 10.1103/PhysRevX.14.041002

[54] P. Guillamat, J. Ignés-Mullol, F. Sagués, PNAS **113**, 5498 (2016). 10.1073/pnas.1600339113 27140604 10.1073/pnas.1600339113 PMC4878504

[55] P. Guillamat, J. Ignés-Mullol, F. Sagués, Mol. Cryst. Liq. Cryst. **646**, 226 (2017). 10.1038/s41467-017-00617-1

[56] É. Fodor, T. Nemoto, S. Vaikuntanathan, New J. Phys. **22**, 013052 (2020). 10.1088/1367-2630/ab6353

[57] J. Walton, G. McKay, M. Grinfeld, N.J. Mottram, Eur. Phys. J. E **43**, 51 (2020). 10.1140/epje/i2020-11973-8 32743686 10.1140/epje/i2020-11973-8

[58] K. Thijssen, D.A. Khaladj, S.A. Aghvami, T.N. Shendruk, PNAS **118**, 38 (2021). 10.1073/pnas.2106038118 10.1073/pnas.2106038118 PMC8463884 34535551

[59] N. Kralj, M. Ravnik, Z. Kos, Commun. Phys. **7**, 222 (2024). 10.1038/s42005-024-01720-8

[60] O. Bantysh, B. Martínez-Prat, J. Nambisan, A. Fernández-Nieves, F. Sagués, J. Ignés-Mullol, Phys. Rev. Lett. **132**, 228302 (2024). 10.1103/PhysRevLett.132.228302 38877903 10.1103/PhysRevLett.132.228302

[61] J. Dervaux, M.C. Resta, P. Brunet, Nat. Phys. **13**, 306 (2017). 10.1038/nphys3926

[62] C. Conklin, J. Viñals, O.T. Valls, Soft Matter **14**, 4641 (2018). 10.1039/C7SM02492D 29796496 10.1039/c7sm02492d

[63] H.R. Vutukuri, M. Lisicki, E. Lauga, J. Vermant, Nat. Commun. **11**, 2628 (2020). 10.1038/s41467-020-15764-1 32457438 10.1038/s41467-020-15764-1 PMC7251099

[64] M. Rajabi, H. Baza, H. Wang, O.D. Lavrentovich, Front. Phys. **9**, 752994 (2021). 10.3389/fphy.2021.752994

[65] M.J. Bowick, N. Fakhri, M.C. Marchetti, S. Ramaswamy, Phys. Rev. X **12**, 010501 (2022). 10.1103/PhysRevX.12.010501

[66] D.K. Sahu, S. Dhara, Soft Matter **18**, 1819 (2022). 10.1039/D1SM01653A 35166748 10.1039/d1sm01653a

[67] V.S. Devika, D.K. Sahu, R.K. Pujala, S. Dhara, Phys. Rev. Appl. **18**, 014030 (2022). 10.1103/PhysRevApplied.18.014030

[68] Y. Kinoshita, N. Uchida, Phys. Rev. E **108**, 014605 (2023). 10.1103/PhysRevE.108.014605 37583184 10.1103/PhysRevE.108.014605

[69] C.Z. van Doorn, J. Appl. Phys. **46**, 3738 (1975). 10.1063/1.322177

[70] J. Kelly, S. Jamal, M. Cui, J. Appl. Phys. **86**, 4091 (1999). 10.1063/1.371333

[71] H.G. Walton, M.J. Towler, Liq. Cryst. **27**, 1329 (2000). 10.1080/026782900423386

[72] D. Svenšek, S. Zumer, Liq. Cryst. **28**, 1389 (2001). 10.1080/02678290110067236

[73] J. Turk, D. Svenšek, Phys. Rev. E **89**, 032508 (2014). 10.1103/PhysRevE.89.032508 10.1103/PhysRevE.89.032508 24730865

[74] D. Svenšek, S. Zumer, Contin. Mech. Thermodyn. **14**, 231 (2002). 10.1007/s001610200088

[75] A.S. Bhadwal, N.J. Mottram, A. Saxena, I.C. Sage, C.V. Brown, Soft Matter **16**, 2961 (2020). 10.1039/C9SM01956A 32119011 10.1039/c9sm01956a

[76] P. Pieranski, F. Brochard, E. Guyon, J. Phys. (Paris) **34**, 35 (1973). 10.1051/jphys:0197300340103500

[77] I.W. Stewart, The Static and Dynamic Continuum Theory of Liquid Crystals (Taylor and Francis, London. N. Y. (2004). 10.1201/9781315272580

[78] M. Grinfeld, M. Langer, N.J. Mottram, Liq. Cryst. **38**, 8 (2011). 10.1080/02678292.2011.588969

[79] N.J. Mottram, J.T. Pinto, G. McKay, Liq. Cryst. **40**, 787 (2013). 10.1080/02678292.2013.783134

[80] F.P. Da Costa, M. Grinfeld, M. Langer, N.J. Mottram, J.T. Pinto, Q. Appl, Math. **70**, 99 (2011). 10.1090/S0033-569X-2011-01265-5

[81] L. Zhao, L. Yao, D. Golovaty, J. Ignés-Mullol, F. Sagués, M. Carme Calderer, Chaos **30**, 113105 (2020). 10.1063/5.0023924 10.1063/5.0023924 33261333

[82] COMSOL. *Multiphysics Version 6.3* (COMSOL, Inc., Burlington, 2025). http://www.comsol.com

[83] P. Pieranski, E. Guyon, Solid State Commun. **13**, 4 (1973). 10.1016/0038-1098(73)90470-5

[84] I. Zúñiga, F.M. Leslie, Liq. Cryst. **5**, 2 (1989). 10.1080/02678298908045422

